# Algorithms for Delivery of Data by Drones in an Isolated Area Divided into Squares

**DOI:** 10.3390/s21165472

**Published:** 2021-08-13

**Authors:** Adrian Marius Deaconu, Razvan Udroiu, Corina-Ştefania Nanau

**Affiliations:** 1 Department of Mathematics and Computer Science, Transilvania University of Brasov, 29 Eroilor Boulevard, 500036 Brasov, Romania; a.deaconu@unitbv.ro (A.M.D.); corina.nanau@unitbv.ro (C.-Ş.N.); 2 Department of Manufacturing Engineering, Transilvania University of Brasov, 29 Eroilor Boulevard, 500036 Brasov, Romania

**Keywords:** drones, network, DTN, mobility schedule, routing algorithms, data delivery

## Abstract

Drones are frequently used for the delivery of materials or other goods, and to facilitate the capture and transmission of data. Moreover, drone networks have gained significant interest in a number of scenarios, such as in quarantined or isolated areas, following technical damage due to a disaster, or in non-urbanized areas without communication infrastructure. In this context, we propose a network of drones that are able to fly on a map covered by regular polygons, with a well-established mobility schedule, to carry and transfer data. Two means exist to equidistantly cover an area with points, namely, grouping the points into equilateral triangles or squares. In this study, a network of drones that fly in an aerial area divided into squares was proposed and investigated. This network was compared with the case in which the area is divided into equilateral triangles. The cost of the square drone network was lower than that of the triangular network with the same cell length, but the efficiency factors were better for the latter. Two situations related to increasing the drone autonomy using drone charging or battery changing stations were analyzed. This study proposed a Delay Tolerant Network (DTN) to optimize the transmission of data. Multiple simulation studies based on experimental flight tests were performed using the proposed algorithm versus five traditional DTN methods. A light Wi-Fi Arduino development board was used for the data transfer between drones and stations using delivery protocols. The efficiency of data transmission using single-copy and multiple-copy algorithms was analyzed. Simulation results showed a better performance of the proposed Time-Dependent Drone (TD-Drone) Dijkstra algorithm compared with the Epidemic, Spray and Wait, PRoPHET, MaxProp, and MaxDelivery routing protocols.

## 1. Introduction

Delay tolerant networks (DTNs) allow communication in environments in which frequent transmission discontinuities are present [1,2,3,4]. They have applications in numerous fields, such as space communication networks [1,2,3], smart cities [5], intelligent transport networks, rural networks, environmental monitoring networks, and vehicle networks [4,6]. Within DTNs, message transmission is based on the store-carry-forward paradigm [7,8]. Devices update their communication routes based on the topological changes of the network, and the mobility of the devices plays an important role [9,10,11].

The role of routing [12,13,14] in DTNs is to find the best path to send data through the network to reach the destination. The routing strategies used by DTNs are classified based on criteria such as connection type between nodes, the time at which the path for messages is established, the amount of information held by the nodes about the network, and the number of copies of a message that a node sends.

Algorithms containing different amounts of DTN information are proposed for investigation in [14]. It has been shown that the performances of these algorithms gradually increase, depending on the amount of information about the network they use. Based on the number of copies of a message, single-copy algorithms (forward based) and multiple-copy algorithms (flood based) have been investigated [15,16]. The Direct Delivery algorithm only sends the message to the destination node, and is suitable for both small- and high-mobility networks, in which the probability of meeting between the nodes is high. Comparisons between classical flood-based protocols, such as Epidemic [17], Spray and Wait [18], PRoPHET [19], and MaxProp [20], were undertaken in [15]. Maximum flow with the static approach in buffer-limited delay tolerant networks was investigated in [21,22].

In public transport networks, the connection type between nodes is based on a well-known schedule and well-defined routes. A DTN composed of pedestrians and cyclists equipped with smart devices was investigated using routing protocols in an Opportunistic Network Environment (ONE) [23]. The MaxProp protocol yielded the best results in terms of delivery probability and average latency. DTN communications in the network storage depend on the store-carry-forward mechanism. A DTN system applied in a communication network on a railway line was found to reduce the message delivery time by 20%, depending on the schedule of trains [8]. In [24], an algorithm to search for the shortest safe path on the network with a time-dependent and edge-length danger factor was proposed. This work is suitable for the optimization of heavy trucks carrying inflammable materials, poison gas, or explosive cargo, and traveling within a city. Based on the simulation in ONE software, the Scheduling-Probabilistic Routing Protocol using History of Encounters and Transitivity (PROPHET) improves the delivery rate and optimizes the delivery delay with low overhead in DTNs for IoT applications [25].

Drone networks are now used in numerous applications across domains including topography [26], aerial observation [27], delivery [28,29], agriculture [30], communications [25,31,32], atmospheric sciences [33,34,35], and rescue missions [36].

Drone flight path planning can be categorized as off-line planning, on-line planning, or cooperative planning [37,38]. The relatively short flight distance of drones due to their limited battery energy [39] can be extended using drone networks.

Flight simulations using a topological map of hexagons, in which each hexagon contains one drone, have shown the potential for application in indoor rescue missions [36]. In [40], coverage path planning with UAVs was studied, addressing simple geometric flight patterns, such as back-and-forth and spiral, and more complex grid-based solutions considering full and partial information about the area of interest. The area was divided into squares and a drone was flown from a square to another. Parcel delivery missions using a drone were simulated based on heuristic flight path planning (HFPP) and other routing algorithms [28]. The results showed that HFPP delivers up to 33% more data packets compared with Encounter-Based Routing and Epidemic routing protocols.

Vehicular ad hoc networks (VANETs), which are a type of mobile ad hoc network (MANET), can be used in an intelligent transport system. VANETs allow the mobile vehicles to establish three main categories of communication: vehicle to vehicle, vehicle to infrastructure, and infrastructure to infrastructure. A specific application of VANET applied to a drone network allows messages to be sent via wireless links [32]. A bio-inspired coordination protocol for a drone flying ad-hoc network (FANET) used for agriculture applications [30] has been used in an ad hoc simulator for a preliminary analysis of the feasibility of drone network design. The performance of the branch and bound search-based mode selection (BBS-MS) for drone-based air-to-ground wireless networks was investigated in [31].

In this study, we investigated six routing algorithms in a network of drones with a mobility schedule to ensure communication between isolated areas or in areas with technical damage. We then qualitatively compared the algorithms. The major contributions of this study are as follows:

- The proposed drone network is independent of the Internet, and is of the DTN type.
- A topological map as a collection of regular polygons (squares) was proposed.
- A time-dependent variant of Dijkstra’s algorithm, which determines the fastest route by taking into account the time when the message reaches the node and the time allocated for data transfer, was developed for the proposed network.
- Five classical algorithms for DTN networks were adapted and tested for the proposed network.
- Simulations based on flight tests were performed to analyze the efficiency of the data transmission. The results obtained by the single-copy and multiple-copy algorithms were compared, in the case of buffer limited capacity.
- Practical application of this work is to facilitate the transmission of information in regions quarantined due to an infectious outbreak, such as COVID-19 pandemic, in regions with technical damage due to a disaster, and in non-urbanized areas without electricity access or communication infrastructure.

The remainder of the paper is organized as follows. In Section 2, a drone network architecture based on the mission profile, and drone sensing and communication, are proposed. Then, the algorithms and protocols for the delivery of data using drones are proposed, described, and analyzed. In Section 3, simulation results are summarized and discussed. Finally, conclusions and possible future work are presented in Section 4.

## 2. Materials and Methods

### 2.1. Drone Network Architecture and Communication

A network map is essential for both drone flight control and simultaneous localization and mission tasks. As noted in [36], three forms of map generation exist: metric, topological, and hybrid maps. A metric map is represented as a grid, geometric, or feature map. Topological maps are represented by graphs comprised of nodes and edges, where nodes represent places, and the edges represent the paths between the nodes [36]. A hybrid map consists of small metric map locations in nodes. These nodes are connected by edges, which are the paths between the metric maps [36]. In this work, a topological map was proposed.

The 2D network surface is intended to be covered with equidistantly distributed points. These points form regular polygons covering the 2D surface. Below, we prove that the only possible means of covering the 2D surface in this manner is by using equilateral triangles or squares. First, we prove the following lemma:

**Lemma** **1.** *There are 3 ways to cover a 2D surface using regular polygons: equilateral triangles, squares, or regular hexagons.*

**Figure 1:**
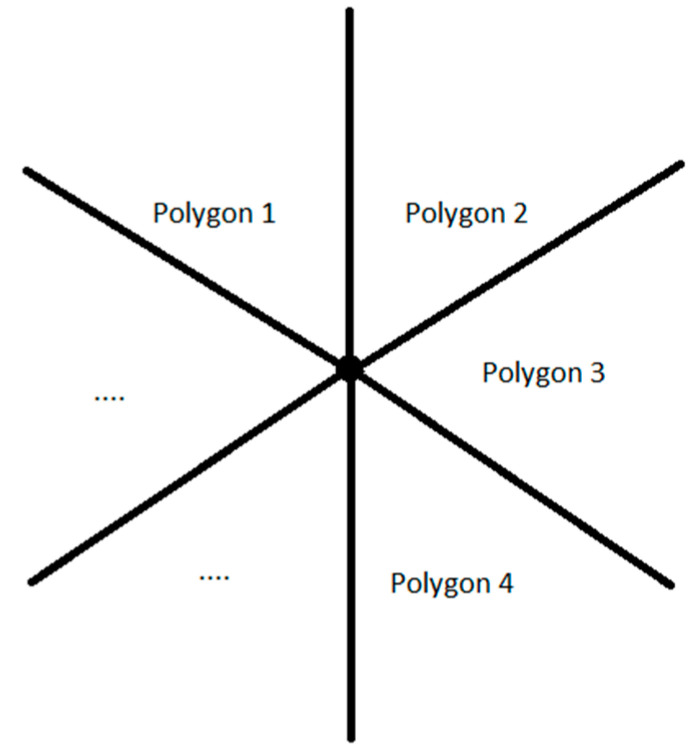
Regular polygons sharing the same vertex.

**Proof** **of** **Lemma** **1.** The sum of the degrees of the angles of a polygon with *n* vertices (*n* ≥ 3) is 180° ⋅ (*n* − 2). Each angle of a regular polygon has $\left[360^{\circ }\cdot \left(n-2\right)\right]/n$ degrees. If a point of the surface is a vertex of a polygon, then it is a vertex for *m* polygons (*m* ≥ 3) around this point (Figure 1). Thus, at such a point we have:

(1) $$m\cdot \frac{180^{\circ }\cdot \left(n-2\right)}{n}=360^{\circ }\Leftrightarrow \frac{2\cdot n}{n-2}=m$$

□

It results that 2⋅n/(n−2)=2+4/(n−2) is an integer value greater or equal to 3. This implies that *n* − 2 is a divisor of 4 and, because *n* ≥ 3, it follows that *n* − 2 is one of the values 1, 2, or 4, which is equivalent to the fact that *n* is 3, 4, or 6, and m is 6, 4 or, respectively, 3. This means that the 2D surface can only be covered with equilateral triangles, squares, or regular hexagons.

**Theorem** **1.** *There are two ways to equidistantly cover a 2D surface with points.*

**Proof** **of** **Theorem** **1.** Using the result from Lemma 1 and the fact that the points are considered equidistant on the 2D surface, it follows that there are only two ways to cover the 2D surface: with equilateral triangles or with squares, because using regular hexagons is impossible (the vertices of the hexagons are not equidistantly positioned). □

**Figure 2:**
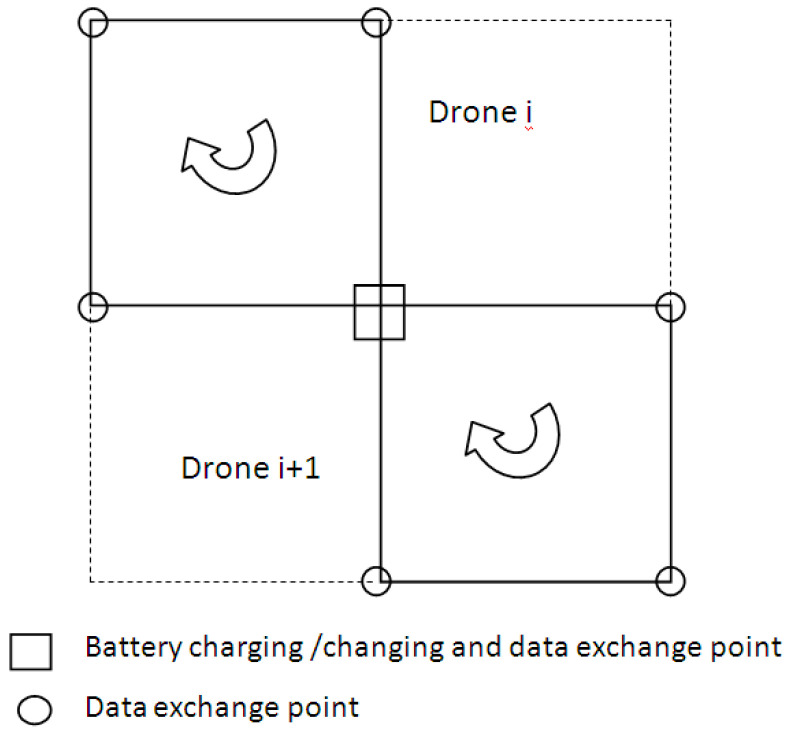
Square cell with two drones.

**Figure 3:**
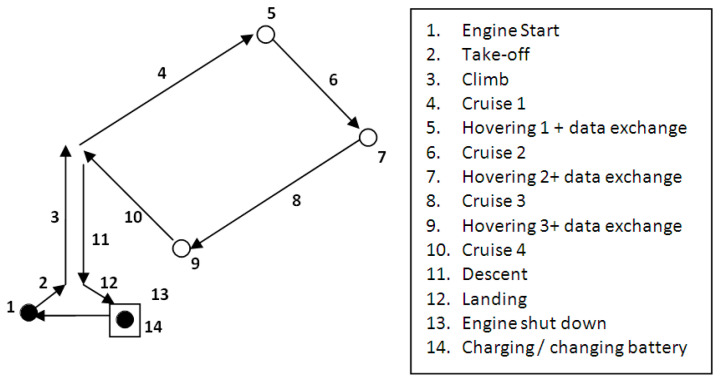
Square shape flight mission profile of the drone.

A network to cover a surface with hexagon cells containing three equilateral triangles was proposed in [41]. In the current paper, a network is proposed to cover a similar surface with square cells containing two operational squares (Figure 2). Each operational cell is covered by a drone. A battery charging/changing dock, which is shared by two drones, is placed in the center of each cell. The docks are able to automatically change/recharge drones, without manual intervention, allowing fully autonomous drone management. The mission profile of each drone consists of several phases or steps: engines start, take off, climb at the cruise altitude, cruise, hovering and data exchange, descent, landing, and engines shut-down. The cruise phase of the flight mission profile consists of four segments (Figure 3). Charging or changing the drone battery is a necessary step at the end of the flight mission.

A drone with a quadcopter configuration [42], i.e., a DJI Mavic 2 Pro (DJI, Shenzhen, China) with a size of 214 × 91 × 84 mm (length × width × height), and takeoff weight of 905 g, was considered in this study. The performance characteristics of this drone are presented in Table 1 [43]. All of the drones of the proposed squares network operate in a pre-programmed manner. The drone network communicates with data exchange points via wireless links.

The endurance of drones can be improved using various methods, such as changing the battery [44,45], charging the battery via wires [46,47], wireless recharging [48], solar cells [49,50], laser-beam in-flight recharging [49,51], and tethered drones [49]. An average time of 90 min is needed to fully charge an empty battery. An automated means of charging a battery via a wire can be performed using a charging platform installed on the ground and a drone retrofit-kit mounted on the drone [46,47]. Thus, the landing gear of the drone is connected by touch with the charging platform after the drone lands, and charging starts automatically. The main disadvantage of this system is that the drone is locked on the ground during the charging of the battery.

An automatic battery changing and recharging system was investigated in [44]. The battery is automatically recharged after it is changed at the station. The electricity needed at each station is able to be provided by a solar panel that charges a battery located at the station. The changing time depends on the efficiency of the changing mechanism, and varies between 15 s [44] and 60 s [45]. In the current study, a maximum changing time before the drone is ready to take off of 60 s was considered.

**Figure 4:**
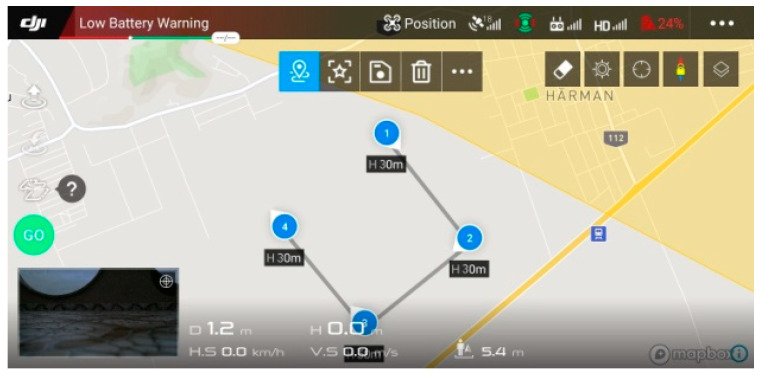
Square-shaped flight mission profile.

Four flight tests were performed based on the square-shaped flight mission in the Brasov area of Romania. The flight tests were performed at a temperature of 2 °C, humidity of 69%, and wind speed of 3.5 km/h. A Samsung S9 smartphone device, on which DJI Go 4 app software (DJI, Shenzhen, China) was installed, connected to a DJI remote controller, was used to program the flight mission segments and to remotely control the drone (Figure 4). A flight altitude of 30 m was chosen [52] and the cruise flight distance of each drone was 12,000 m.

The theoretical flight times calculated based on the drone specifications (max. ascent, descent speeds, and cruise speed) are not realistic for simulations. The main factors that influence the experimental flight times are acceleration and deceleration of the drone, and wind speed. The mean flight time for each flight segment was calculated based on the flight tests (Table 2). Moreover, the average calculated cruise speed of the drone obtained from the flight tests was 12.93 m/s.

The percentage of the remaining drone battery obtained at the end of the flight mission was 16% for the rectangular cell. The charging time for the drone battery was 79 min. A safety multiplier of 1.1 was applied to obtain the considered charging time, and the resulting time was used in the simulations. Thus, 87 charging minutes were considered for the rectangular cell flight. The average values of the flight times for each segment were used as input parameters for the simulation of the drone networks in the DTN algorithms.

Eight high-resolution and two infrared sensors were used on the DJI Mavic 2 Pro. These sensors enabled omnidirectional obstacle sensing, to determine the relative speed and distance between the drone and the object, and to ensure good stability in forward and hovering flight. Left, right, up, down, forward, and backward obstacle sensing were used.

**Figure 5:**
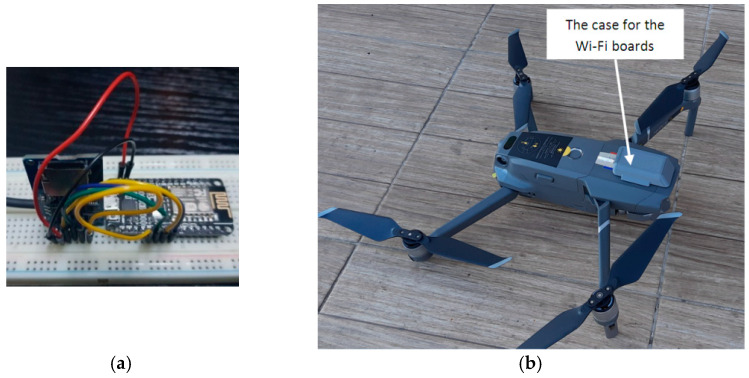
(**a**)Testing stand of the NodeMCU Lua Wi-Fi board and the micro-SD card module mounted on a testing breadboard. (**b**)The drone with the 3D-printed case and the Wi-Fi boards mounted on it.

A NodeMCU Lua Wi-Fi, V3, ESP-12E, CP2102 Wi-Fi Arduino development board (Espressif Systems, Shanghai, China) was used for the data transfer between the drones and the stations. The main characteristics of the NodeMCU are shown in Table 3. The data were stored on a micro-SD card. The Wi-Fi board and micro-SD card module are very lightweight and have very low power consumption. The experimental layout, consisting of the NodeMCU Lua Wi-Fi board and the micro-SD card module mounted on a breadboard, is shown in Figure 5a. Arduino code was used to program the Wi-Fi boards.

A protective mounting case (Figure 5b) for the Wi-Fi boards was designed using the SolidWorks version 2016 software (Dassault Systèmes, Waltham, MA, USA), and then 3D printed using material extrusion technology from PLA (BCN3D Technologies, Barcelona, Spain) on a BCN3D Sigma R19 printer (BCN3D Technologies, Barcelona, Spain).

The Wi-Fi board, micro-SD module, SD card, connection wires, and power cable weighed a total of 21.8 g, thus representing an increase of 2.4% in the total weight of the drone.

### 2.2. Algorithms and Protocols for Delivery of Data Using Drones

The DTN was modeled with a graph having fixed and mobile nodes. There are no connections between the fixed nodes, so there is no possibility of direct data transmission because the considered distance between the nearest two nodes is 3000 m. Network connections are provided by mobile nodes (drones), but their condition is not always the same; they have periods when they are active and periods when they are inactive. This means that there is not always an end-to-end path available between any two nodes in the graph.

**Figure 6:**
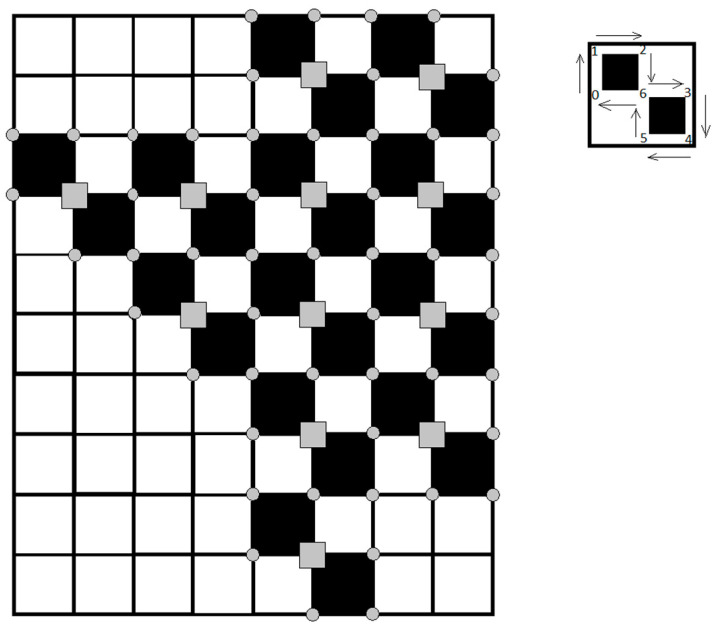
Squares network considered for experiments.

We tested five well-known routing algorithms for DTNs (Epidemic, Spray and Wait, PRoPHET, MaxProp, and MaxDelivery [54]), and a newly proposed TD-Drone Dijkstra approach, on the square-shaped network shown in Figure 6. We performed tests on the network by choosing random sources and random destinations that could be located on any node (marked as a gray circle or a gray rectangle). We considered the drones worked each day from 7:00 a.m. to 6:00 p.m. and the messages may leave a source node between 7:00 a.m. and 5:00 p.m. We considered 1000 messages randomly sent within this interval of time.

Epidemic is the basic form of a flood-based routing protocol: when two nodes meet, they identify the packages that the other node has and transfer the packages that it does not have. At the end of the process, the two nodes have the same content in the buffer. This process is repeated each time two nodes come into contact. When a node has a copy of a message, it waits to meet the destination. In this case, the resource consumption is high but, in a high mobility network, the delay of message transmission is small. In the current network configuration, the algorithm produces poor results due to the low number of contacts between nodes.

Spray and Wait is an algorithm with two phases, one for sending messages (spray) and one to wait for the contact with the destination node (wait). This algorithm circulates in two variants—standard and binary—depending on the number of spread copies of the message. It acts in the same manner as Epidemic, with an important difference: the number of spread copies is constant. The spray phase of the standard approach consists of spraying L copies of the message by the source node itself. The spray phase of the binary approach consists of spraying half of the number of copies to a meeting node. In this case, not only does the source spray messages, but also every node that has more than one copy. The nodes that have only one copy enter the wait phase. This algorithm has the disadvantage that nodes must keep track of other nodes’ movement, but the advantage is that the level of flooding is limited.

PRoPHET is similar to Epidemic, with the exception that it uses information from the buffer of the other node to update its predictability vector. Each node calculates the predictability of the message delivery and sends the message onward only if the contact node has higher predictability than its own. The disadvantage of this approach is the relationship between the overhead ratio and the number of nodes—as the number of nodes increases, the overhead ratio increases [15]. This protocol is known for the complexity of its forwarding strategy. Thus, it consumes a significant quantity of resources to process and store historical values. This approach is feasible for networks with high computation and infrastructure capabilities.

MaxProp is an algorithm based on prioritizing packet transmission and discarding. The packets in the queue are divided into two categories: those below the “n” hop threshold (up to that point), and those above this threshold. Newer packages that have not traveled too much are considered a priority and the guarantee that they will reach their destination is considered to be high. This algorithm also requires high computation and infrastructure capabilities. This protocol has low performance when nodes have small buffer sizes because of the adaptive threshold calculation, but gives better performance with a larger buffer size. It has a so-called slow start problem, because, in the case of a big network, it may take a very long time before each node receives the delivery predictability of other nodes because of the disconnecting nature of the networks, as shown in [20].

MaxDelivery [54] is an algorithm based on prioritizing message delivery using an appropriate buffer management strategy that consists of a forwarding, dropping, and buffer-cleaning mechanism.

Next, we propose our time-dependent Dijkstra algorithm to find a route between two nodes in our drone network. Dijkstra’s algorithm is used to find the shortest path connecting two nodes in a network [55]. Our routing problem can be modeled using a time-dependent oriented network [56] defined as follows.

**Definition** **1.** *A triple G = (V, A, f) is called a time-dependent oriented network, where V is a set of vertices, A ⊆ V × V is a set of arcs, and f is the time dependency function defined on each arc, f: A × T → T, T ⊆ R+ is called the time set and when moving on the arc a = (u, v) ∈ A from node u to node v, f(a, t) ∈ T is the moment of arrival at node v if node u is left at the moment t ∈ T. Of course, f(a, t) > t, for each arc a ∈ A and for any moment t ∈ T.*

In our problem, V is the equidistantly distributed set of 2D points, A is the set of connections between these points ensured by drones, and T = {0, 1, 2, …} is the set of counting seconds in a day. The value of f(a, t) must be computed as quickly as possible. Thus, for each arc a = (u, v), the arrival moments of drones at node v are maintained in order to enable a binary search to be undertaken when f(a, t) is calculated at arc a and time t. The arrival moments of drones for all arcs are pre-calculated (once before starting to use the algorithm) because an exact schedule of drones is known based on each drone’s starting second in a day, its travel on each arc, its data transfer at nodes, and its wireless charge/battery change time.

**Figure 7:**
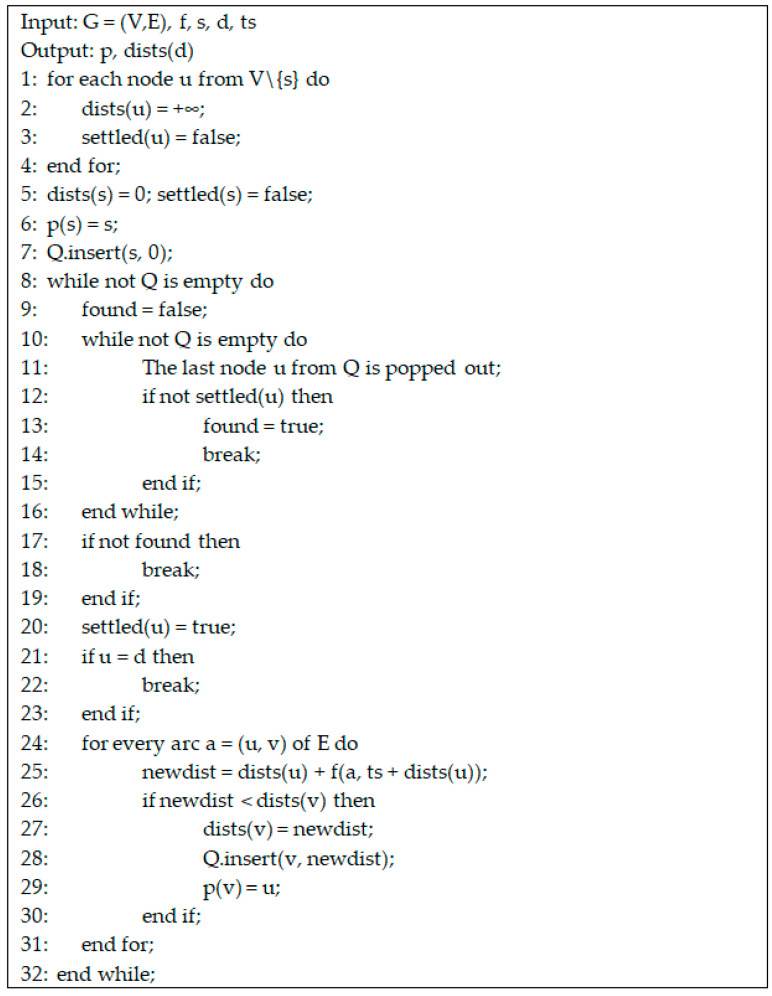
Pseudo-code of TD-Drone Dijkstra algorithm.

For a given source node s ∈ V, the function dists: V × T → N is introduced, where dists(u, ts) is the distance in seconds from s to u starting from the moment ts, i.e., it is the minimum number of seconds needed to go from node s to node u ∈ V on the arcs of A if source s is left at moment ts. Of course, dists(s, ts) = 0 for every ts ∈ T. If node u is not reachable (accessible) from s, then dists(u, ts) = +∞. For a given destination node d, our problem is to determine dists(d, ts) at a given time ts. The pseudo-code for the time-dependent Dijkstra algorithm is presented in Figure 7.

**Figure 8:**
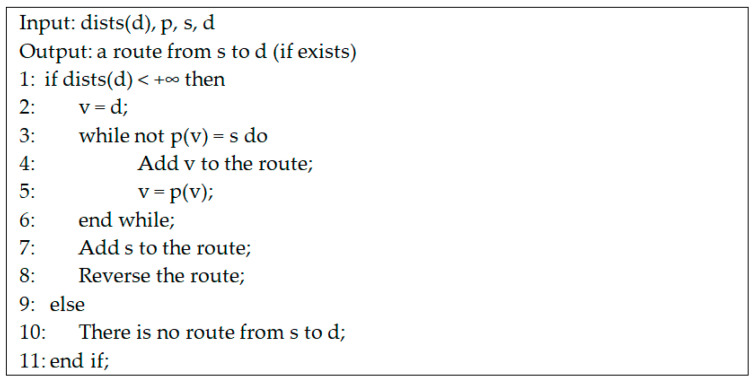
Pseudo-code for the construction of the route after the execution of the TD-Drone Dijkstra algorithm.

Q is a priority queue. This means that, at any moment, the nodes from Q are sorted in ascending order according to their distance in seconds from s. At the end of the algorithm, if destination d was reached, the values kept in the so-called predecessor vector p are used to determine the route from s to d. If a node v was reached, then this was done using the arc (p(v), v), where node p(v) is called the predecessor of v. After the time-dependent Dijkstra algorithm is executed using the staring moment ts, the route from s to d (if it exists, i.e., dists(d) < +∞) is determined using the algorithm presented in Figure 8. Because the nodes of the route are found by the above algorithm in inverse order (from d to s), the route must be reversed at the end.

For each data file that has to be delivered, a “json” file is attached that stores all of the information needed to transfer the file from the source to the destination: delivery type (Dijkstra, Epidemic etc.); file information (name, size, time-to-leave); route information (node codes: stations and drones); etc. The json files that store message information for the algorithms Epidemic, Spray and Wait, PRoPHET, MaxProp, and MaxDelivery implemented in the ONE environment are similar to those considered in [41]. Each route starts and ends with a station id. Each station id (except for the destination) is followed by a drone id, and each drone id is followed by a station id. When a drone arrives at a station, a transfer is initiated between the drone and the station. The drone transfers to the station all of the files that have the station’s id in the attached json files. After the drone transfer to the station is completed, the station transfers to the drone all of the files that have the drone’s id in the json file.

**Figure 9:**
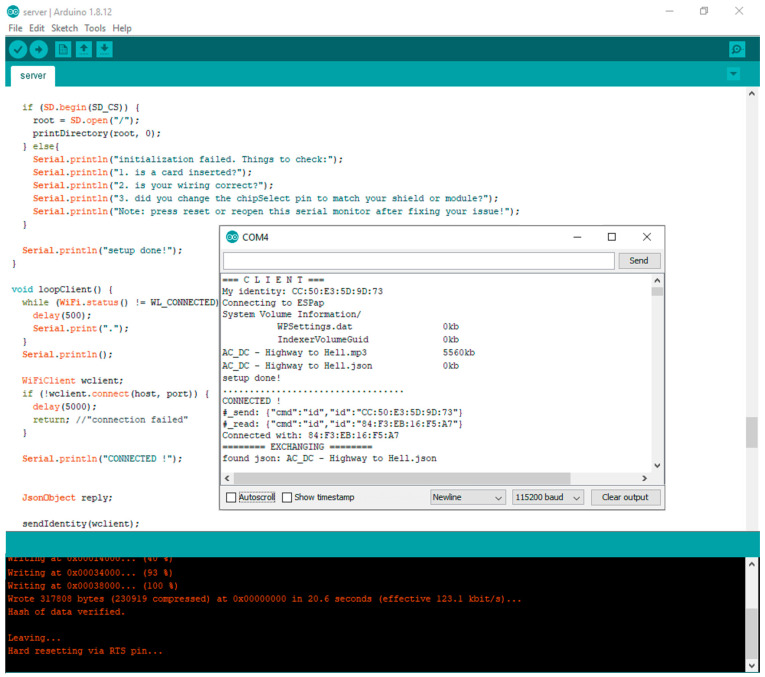
The console output of the Wi-Fi board.

The range of the Wi-Fi boards was tested. The connections between the boards and file transfer were performed at a distance of up to 85 m with no obstacles in between. In Figure 9, the console output of the Wi-Fi board is presented. The following steps are executed: setup (upload speed is set to 115,200, MAC address is obtained), the connection between two Wi-Fi boards is established, the file transfer is performed, and, finally, the Wi-Fi boards are disconnected.

In our model, when transferring files, the drone hovers over the station at a height of 30 m, which is significantly less than the maximum distance obtained in range tests. The transfer speed, including writing on and reading from the SD card, was also tested at a distance of 30 m. An average of 5.81 Mbps was obtained, which means that a file of 10 MB was transferred in 13.77 s.

## 3. Simulation Results and Discussion

The proposed network of drones can be applied in various scenarios, such as in remote quarantined or isolated areas, following technical damage due to a disaster (e.g., an earthquake), or in non-urbanized areas without electricity access or communication infrastructure. For instance, a remote quarantined zone (e.g., due to the COVID-19 pandemic), in which buildings are separated by a safe distance, is considered. In this scenario, each building may be a house in which patients are isolated, a warehouse storing food or drugs, a laboratory in which medical tests are performed for patients, or a location at which doctors are working (isolated from patients). Data packages (medical images, tests, results, prescriptions from doctors, etc.) must be sent between these buildings. The communication between these buildings can be achieved by drones organized in a square-shaped network.

To validate the proposed method, simulations were performed using the drone network maps as a collection of squares. Most of the simulations were performed using the Java-based simulator ONE and its provided facilities, using an ASUS ROG GL752VW-T4015D laptop with Intel^®^ Core™ i7-6700HQ 2.60GHz processor (Asus, Taipei City, Taiwan), and 8 GB of RAM. The routing protocols used for the simulation within the ONE simulator were Epidemic, Spray and Wait, PRoPHET, MaxProp, and MaxDelivery.

**Figure 10:**
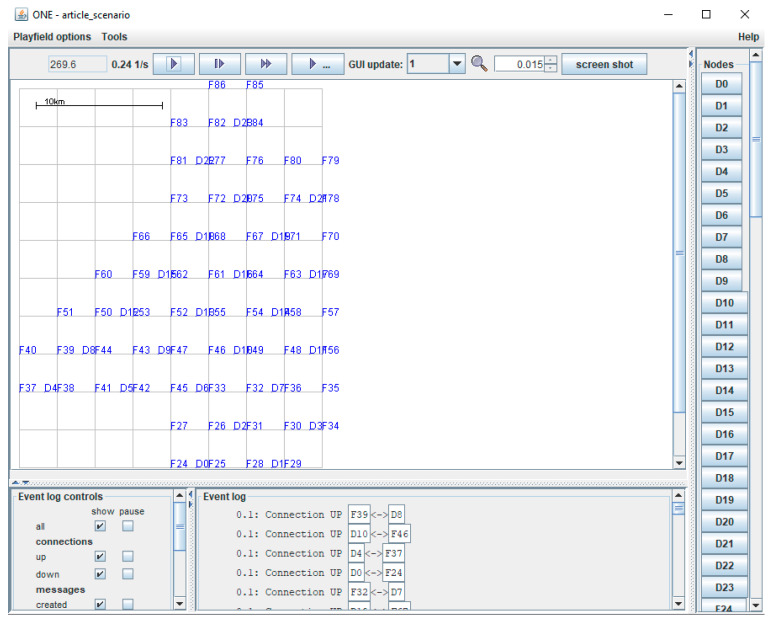
Design of the squares network in the ONE simulator.

The following main steps were used for implementation of the ONE scenarios. The first step consists of defining the map (Figure 10) in wkt file format, in which the coordinates of all the points on the map, including points that establish the route of each drone, are defined. The initializations of the algorithms that define the mobility of drones consist of establishing the initial positions of drones and the recharging/changing points, associating each drone with a recharger/changing point, establishing the stationary points for data transfer, and defining the route of each drone. The final step is the establishment of the simulation parameters, as shown in Table 4. The time parameters, such as the travel autonomy time, the hovering time for the transfer points, and the parking time at the charging or changing points, were established based on the experimental flight tests of the DJI Mavic 2 Pro drone.

The proposed time-dependent Dijkstra variant was implemented in Visual C++ 2017 programming language. The application has about 1100 lines of C++ source code.

The application written in C++ was executed for each of the two considered situations: squares with battery charging, and squares with battery changing. For each case, the same 1000 route simulations considered in the ONE experiments were executed. 

The delivery rate and latency metrics were used to measure the performance of all six routing protocols analyzed in this paper. The delivery rate is determined as a ratio between the number of delivered messages and the number of created messages. The latency is the average time needed for a message to reach the destination starting from the source (departure node).

Detailed results of the comparison between changing the battery and charging the battery cases are presented in Table 5. The results obtained in this paper by simulation were compared with those obtained in [41].

**Figure 11:**
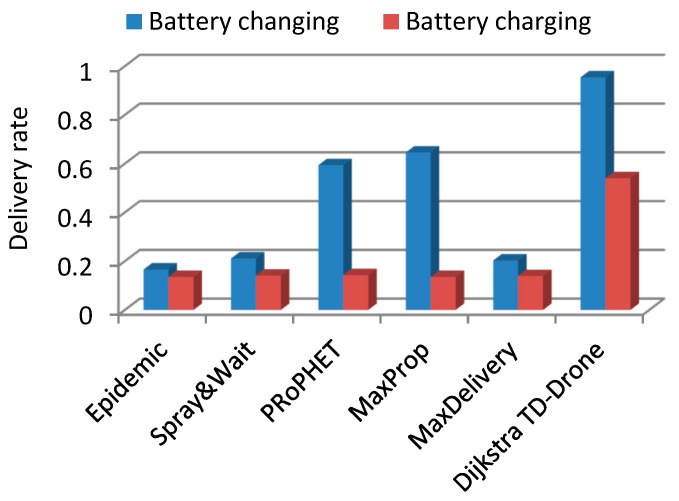
Delivery rate in the squares drone network.

**Figure 12:**
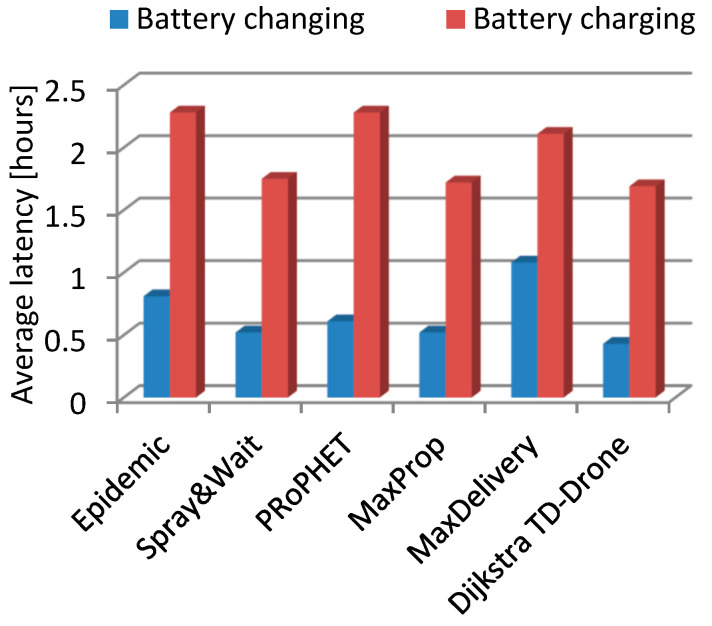
Average latency in the squares drone network.

In the case of the squares network and battery changing, the values of the delivery rate were within the range of 0.166 to 0.646 for the routing protocols Epidemic, Spray and Wait, PRoPHET, MaxProp, and MaxDelivery. The best delivery rate was 0.954 for the TD-Drone Dijkstra protocol (Figure 11). The worst result in terms of latency was obtained for MaxDelivery, and TD-Drone Dijkstra’s average latency was the best, as expected (Figure 12).

The delivery rate in the case of drone battery charging was between 0.135 and 0.143 for the routing protocols Epidemic, Spray and Wait, PRoPHET, MaxProp, and MaxDelivery. The maximum delivery rate was 0.54 and was obtained for the TD-Drone Dijkstra protocol. These low values were obtained because of the large delay due to the battery charge. The worst results for latency were obtained for PRoPHET and Epidemic, and TD-Drone Dijkstra’s latency was also the best.

The comparison between average latencies was performed on the routes where the delivery was successful for all of the algorithms (66 routes for the case of battery change and 64 routes in the case of battery recharge).

The best delivery rate in all cases was given by the TD-Drone Dijkstra algorithm because its buffer load was the lowest (the data was loaded only in the stations and drones belonging to the calculated route). The latency in the case of Dijkstra’s algorithm was high because it can deliver most of the packages. Generally, the other algorithms can deliver only on the shorter distances due to buffer restrictions. It is known that Dijkstra’s algorithm results in the shortest path, and, therefore, the best latency.

**Figure 13:**
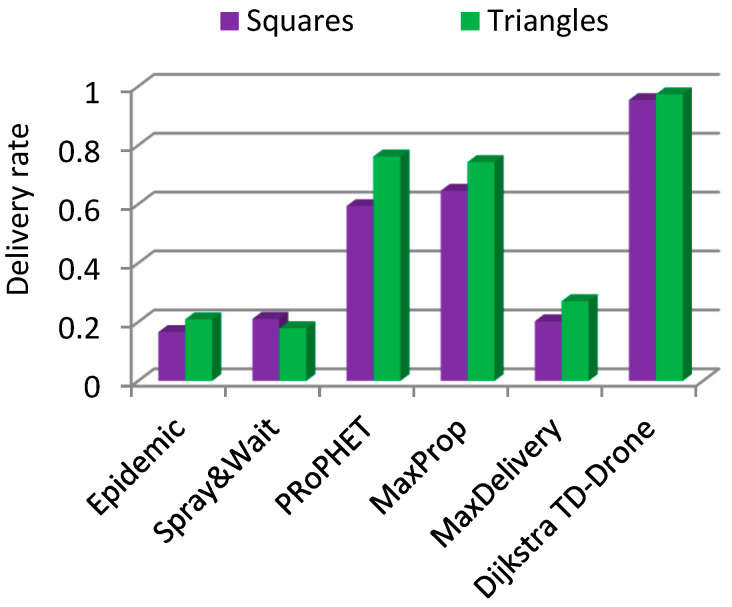
Delivery rate, battery changing, squares vs. triangles.

**Figure 14:**
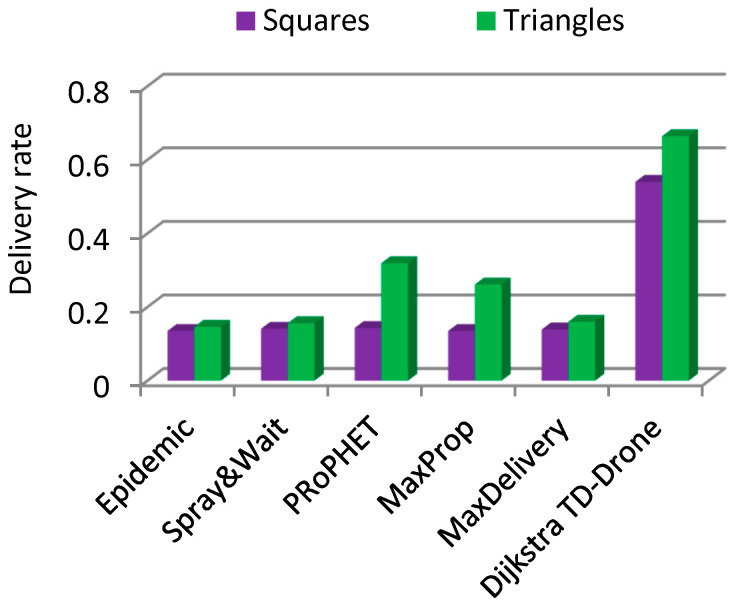
Delivery rate, battery charging, squares vs. triangles.

The delivery rate was better for the triangles drone network (Figure 13 and Figure 14), with the exception of the Spray and Wait algorithm in the battery changing case. For PRoPHET, Max Prop, and TD-Drone Dijkstra in the battery charging case, the latency was significantly better.

**Figure 15:**
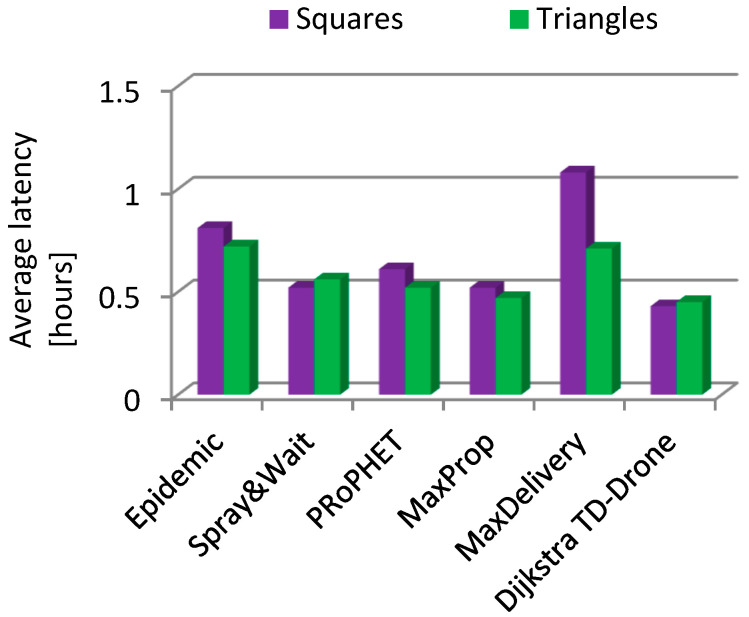
Average latency: battery changing, squares vs. triangles.

**Figure 16:**
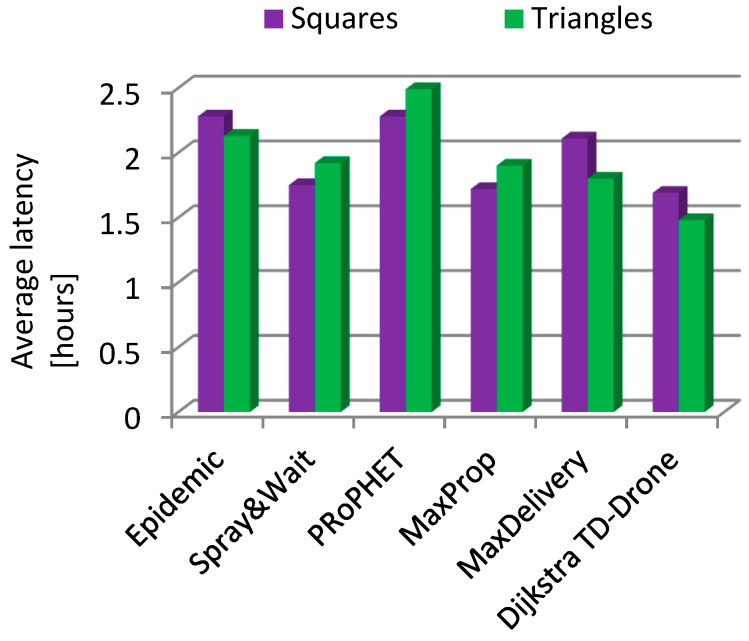
Average latency: battery charging, squares vs. triangles.

Latency in the case of changing the battery in the triangles drone network was generally better (Figure 15), with the exception of Spray and Wait, and in the case of Max Delivery a significantly better latency was obtained. In the case of battery charging (Figure 16), latency was better in the triangles drone network for three algorithms (Epidemic, Max Delivery, and TD-Drone Dijkstra), and, for the other three algorithms, the latency was better in the squares drone network.

In essence, the delivery rate was considerably better for the triangles drone networks, and latency was generally better for the triangles case. The significant disadvantage of the triangles drone network is that double the number of drones is needed to cover approximately the same surface, and double the number of loading/changing stations is required, although the total number of fixed communication stations is similar (63 vs. 65).

Epidemic, Spray and Wait, PRoPHET, and MaxProp are classic algorithms used for DTN. However, in our case, as shown, the time-dependent Dijkstra algorithm adaptation can be successfully used because since the flight timetables are known. There are numerous advantages of the TD-Drone Dijkstra algorithm: an exact and optimum route is a priori calculated, ensuring the fastest time of delivery from departure to destination if the route exists; a message is not unnecessarily sent in the network if no route exists from departure to destination; multiple copies of the messages are not unnecessarily spread through drone and station buffers, resulting in unnecessary overloading of the buffers; and, finally, the rate of delivery success is maximized. The drawback of the TD-Drone Dijkstra algorithm is that the route is calculated using the information about the operating drones and stations at the moment of route calculation and, if a drone or a station from the route is down on this route, the message does not reach the destination. All the routes passing through the station or drone that is down are compromised until the fault is detected. Moreover, this problem reappears when the drone/station is fixed until the moment this information is updated. However, the chance of this problem occurring is low and, if it appears, it may be fixed in time following the repair of the drone/station or when the current status of the network is updated. Using the example of the Spray and Wait or Epidemic algorithms presented in this paper, any message has the chance to reach the destination even if drones or stations are down because a copy of the message is spread in the network.

## 4. Conclusions and Future Work

This paper presents a novel method of communication in quarantined or isolated areas, or areas with technical damage, using networks of drones that fly based on a well-established mission plan and schedule, on 2D surfaces covered by squares. Two situations of drone battery management—charging and battery changing stations—were investigated.

A network of square cells with two drones in each cell was proposed to cover a geographical area. The drone network was simulated based on input data from experimental flight tests of a quadcopter using six routing algorithms.

A TD-Drone Dijkstra algorithm (single-copy algorithm) and multiple-copies algorithms were proposed to simulate a Delay Tolerant Network of drones. Results showed a delivery rate ranging from 0.166 to 0.954 in the drone network with battery changing, and from 0.135 to 0.540 in the drone network with battery charging. The best latency of 0.43 h for a drone network with battery changing was obtained using the TD-Drone Dijkstra algorithm. Thus, the best results were obtained for the TD-Drone Dijkstra algorithm, which was able to deliver most of the data packages in the shortest time.

The traditional DTN algorithms, such as Epidemic, Spray and Wait, and MaxProp, produced lower results due to the small number of contacts between nodes, and a low number of message exchanges. The fastest communication was established for the drone squares network with battery changing. It was found that the battery change scenario led to an increase in the delivery rate of 76% compared to the battery charge scenario.

It was found that the battery change scenario led to an increase in the delivery rate of ~200% compared to the battery charge scenario.

The fastest communication was found for the drone triangular network with battery charging, and for the drone square network with battery changing. However, the drone square network is considerably cheaper than the drone triangular network. Thus, if cost is not an issue, the triangle network of drones may be implemented because better performances can be achieved. However, if there is a budget constraint, then the square network type is more suitable.

In future work, we will aim to apply this network of drones to parcel delivery during emergencies in remote quarantined zones.

## Figures and Tables

**Table 1:** Drone performance [43].

Parameter	Value
Max ascent/descent speed	4 m/s; 3 m/s
Max flight time (no wind)	31 min (at a consistent 25 km/h)
Max flight distance (no wind)	18 km (at a consistent 50 km/h)
Drone battery	3850 mAh, 1800 mA, 3.83 V

**Table 2:** Flight test results.

Mission Phase	Mean Flight Time	Standard Deviation
Take off + climb (30 m)	8.24 s	0.193
Cruise segment (3000 m)	232 s	0.187
Descent + landing (30 m)	12.12 s	0.085
Transfer data (3 points)	120 s	-
Total flight on square cell	1308 s	0.651

**Table 3:** Wi-Fi NodeMCU main characteristics [53].

Parameter	Value
ESP8266 chip	26 MHz, 4 MB flash, 160 KB RAM
Dimensions (L × W)	48 mm × 25 mm
Operating temperature	−40 °C to + 125 °C
Weight	8 g

**Table 4:** Simulation parameters for the square-shaped flight mission.

Parameter	Value
Number of drones for cruise	24
Number of fixed transfer points	63
Number of charging/ changing battery points	12
Average cruise speed of a drone	46.55 km/h (12.93 m/s)
Flight height of drones	30 m
Operating time of the drone in one day	11 h
Data transmission speed	2 Mbps
Drone buffer space	2 Gb
Message size	500 kb–1 Mb
Message time to live	10 h
Source and destination of messages	any station
No. of route simulations	1000

**Table 5:** Efficiency factors in drone network.

Algorithm	Delivery Rate				Latency (hours)			
	Battery Changing		Battery Charging		Battery Changing		Battery Charging	
	Squares	Triangular	Squares	Triangular	Squares	Triangular	Squares	Triangular
Epidemic	0.166	0.209	0.135	0.146	0.81	0.72	2.28	2.13
Spray and Wait	0.211	0.179	0.141	0.156	0.52	0.56	1.75	1.92
PRoPHET	0.594	0.762	0.143	0.319	0.61	0.52	2.28	2.49
MaxProp	0.646	0.743	0.135	0.261	0.52	0.47	1.72	1.90
MaxDelivery	0.203	0.271	0.139	0.160	1.08	0.71	2.11	1.80
TD-Drone Dijkstra	0.954	0.973	0.540	0.664	0.43	0.45	1.69	1.48

## Data Availability

Not applicable.
